# Electrical Stimulation Therapy – Dedicated to the Perfect Plastic Repair

**DOI:** 10.1002/advs.202409884

**Published:** 2024-12-16

**Authors:** Kexin Deng, Ruizeng Luo, Ying Chen, Xiaoqiang Liu, Yuanyin Xi, Muhammad Usman, Xupin Jiang, Zhou Li, Jiaping Zhang

**Affiliations:** ^1^ Department of Plastic Surgery State Key Laboratory of Trauma and Chemical Poisoning Southwest Hospital Third Military Medical University (Army Medical University) Chongqing 400038 China; ^2^ Beijing Institute of Nanoenergy and Nanosystems Chinese Academy of Sciences Beijing 101400 China; ^3^ School of Nanoscience and Engineering University of Chinese Academy of Sciences Beijing 100049 China; ^4^ A Breast Disease Center Southwest Hospital Third Military Medical University (Army Medical University) Chongqing 400038 China; ^5^ Department of Plastic Surgery and Burn Central Hospital Affiliated with Chongqing University of Technology Chongqing 400054 P.R. China

**Keywords:** cellular activity, clinical therapy, electrical stimulation, nanogenerator, tissue repair

## Abstract

Tissue repair and reconstruction are a clinical difficulty. Bioelectricity has been identified as a critical factor in supporting tissue and cell viability during the repair process, presenting substantial potential for clinical application. This review delves into various sources of electrical stimulation and identifies appropriate electrode materials for clinical use. It also highlights the biological mechanisms of electrical stimulation at both the subcellular and cellular levels, elucidating how these interactions facilitate the repair and regeneration processes across different organs. Moreover, specific electrode materials and stimulation sources are outlined, detailing their impact on cellular activity. The future development trends are projected from two perspectives: the optimization of equipment performance and the fulfillment of clinical demands, focusing on the feasibility, safety, and cost‐effectiveness of technologies.

## Graphical abstract

This review discusses various types of electrical stimulation sources and materials, delving into the roles and mechanisms of electrical stimulation therapy in plastic surgery for tissue repair. This work broadens the clinical perspective of electrical stimulation, covering diverse repair needs from bone to soft tissues, and provides insights into future advancements in equipment optimization and clinical applications.

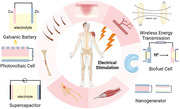

## 1 Introduction

Plastic repair aims to reconstruct and repair the damaged tissues with many types of treatments,^[^ ^1^ ^]^ including various tissue grafting. Perfect plastic repair requires not only the preservation of the aesthetic integrity of the tissue but also the restoration of normal physiological functions in the affected area, such as skin sensation, motor function, and other related physiological characteristics. Current tissue grafting methods have many limitations, including restricted donor availability, rejection reactions, poor vascularization, inadequate tissue integration, and infection risks. These issues highlight the importance of using non‐invasive methods to induce in situ tissue regeneration.

Endogenous electric fields refer to the naturally generated electrical signals within living organisms. These electrical signals, which arise from the physiological activities of tissues and cells, are crucial for various physiological and pathological processes, including cardiac electrical activity, neural signal transmission, and muscle contraction. They are closely linked to metabolism, ion channel dynamics, and neuromuscular excitability.^[^ ^2^, ^3^ ^]^ Endogenous electric fields can enhance intercellular signaling by regulating the membrane potential and the activity of ion channels, thereby promoting cell behavior (such as migration, proliferation, and differentiation) during tissue regeneration, providing a biological basis for the application of electrical stimulation therapy in tissue repair and regeneration.^[^ ^4^ ^]^

Electrical stimulation therapy utilizes electrical signals generated by external sources to regulate physiological activity in vivo.^[^ ^5^ ^]^ The earliest electrical stimulation therapy equipment relied on external power sources and used bulky electrodes applied to the skin surface, resulting in cumbersome devices that limited patients’ daily activities. With advancements in technology, electrical stimulation devices have gradually evolved toward miniaturization, portability, and intelligence.^[^ ^5^, ^6^ ^]^ Moreover, in the field of wound repair, electrical stimulation promotes cell migration and proliferation. In nerve repair, it aids in activating nerve regeneration. For muscle repair, electrical stimulation enhances muscle contraction, while in bone repair, it stimulates the activity of osteoblasts. Due to these advantages, electrical stimulation is widely applied in tissue regeneration, significantly improving the success rate of reconstructive surgeries.

This review deeply explores the current development status of exogenous electrical stimulation therapy, summarizes its mechanism and detailed application, especially in tissue transplantation technology. Initially, we list various types of exogenous electrical stimulation devices and sources, focusing on the progress of power supply and electrode material research, and show their potential for integration into clinical devices. Subsequently, the cellular activities and molecular mechanisms regulated by electrical stimulation are introduced in detail, laying the biological foundation for its application in tissue repair. Its application in various tissue repairs is highlighted and the current challenges and future development directions of this therapy in clinical practice are discussed.

## 2 Exogenous Electrical Stimulation Therapy Technology

**Figure 1:**
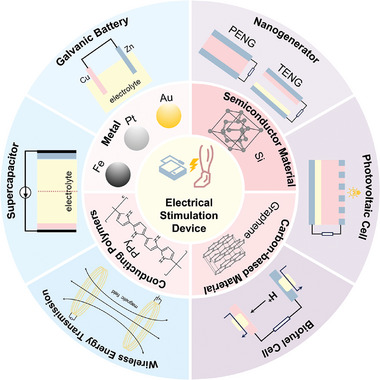
Power supply and electrode selection strategy of electrical stimulation therapy equipment.

Exogenous electrical stimulation technology, as an innovative tissue engineering strategy, has been widely used in clinical treatment due to its simple operation, non‐invasive or minimally invasive nature, and adjustable stimulation parameters. In many applications of exogenous electrical stimulation technology, power supply, and stimulation electrodes play a core role. They are key components for generating and transmitting electrical stimulation signals, which directly affect the effectiveness and safety of treatment (**Figure** **1**). With the continuous advancement of engineering technology and the growing demand for electrical stimulation technology in clinical treatment, traditional electrical stimulation devices that rely on large, expensive and non‐portable devices have gradually been replaced by new and innovative devices. The diversity of electrode materials and characteristics enables them to meet the needs of different application scenarios and disease treatment. The development of miniaturized power supplies and the innovation of electrode materials have made the application prospects of exogenous electrical stimulation technology in clinical treatment more and more broad. They not only provide doctors with more treatment options, but also bring patients hope for a higher quality of life, indicating an important development direction for personalized and precision medicine in the future.

### 2.1 Power Supply of Electrical Stimulation

Traditional electrical stimulation therapy equipment generates electrical output by utilizing power sources that create various forms of current through rectification, filtering, frequency modulation, and other methods. The built‐in power supply is direct current (DC) power (output direct current of different strengths) and signal generator (output pulse current (PC) of different frequencies, strengths, and pulse width). Although traditional electrical stimulation devices have been very effective in treating diseases, their importability and high cost have resulted in huge medical expenses. In the current trend of developing personalized medical services, the needs of clinical treatment have raised new requirements for electrical stimulation therapy sources. As a result, the development of wireless/passive, miniaturized, low‐cost, portable, safe, and stable electrical stimulation therapy equipment has become a prominent topic.

#### 2.1.1 Galvanic Battery

Some researchers are considering using small power sources, such as galvanic batteries, instead of DC power sources due to the large and expensive nature of traditional power sources. Various portable electronic devices widely use a galvanic battery, a common mobile power supply that generates current through the oxidation and reduction reactions of two different metals in the electrolyte.^[^ ^7^ ^]^ The galvanic cells have the characteristics of small size, portability, and high energy conversion efficiency.^[^ ^8^ ^]^ Galvanic batteries have limited energy storage, requiring replacement once the battery runs out. Rechargeable galvanic batteries solve the problem of frequent battery replacement and are the most common power supply in miniaturized electrical stimulation equipment at present.^[^ ^9^ ^]^ The power management module enables it to meet the various requirements of electrical stimulation therapy. Its rechargeable characteristics make it have a long service life. In addition, based on the working principle of galvanic cells, researchers applied body fluids as electrolytes and biocompatible metals (such as Mg, Fe, Zn, etc.) as electrodes to construct galvanic cells in the original location of the tissue for electrical stimulation.^[^ ^10^ ^]^ However, the voltage of the galvanic cell is low, and it needs to rely on a large and complex power management module when used for high‐intensity electrical stimulation, which greatly increases the cost of electrical stimulation therapy.

#### 2.1.2 Supercapacitor

A supercapacitor, an electrochemical capacitor with a high energy density, stores electric energy by forming a double electric‐layer capacitor through charge separation on the interface between the electrolyte and electrode.^[^ ^11^ ^]^ Compared with galvanic cells, supercapacitors have higher power density and cycle life. Moreover, supercapacitors use the physical process of double‐layer charge and discharge or the fast/reversible chemical process on the surface of the electrode material to recharge much faster than the galvanic battery.^[^ ^12^ ^]^ Most supercapacitors, due to their high concentration of cytotoxic electrolytes, are not suitable for body implants. To meet the needs of medical devices that can be implanted with electronics, scientists have created supercapacitors that use Mo foils, MoOx sheets, and Alg‐Na gel electrolytes to control how quickly the batteries break down. The supercapacitor is degradable and has good biocompatibility, which can power implantable electronic devices.^[^ ^13^ ^]^

#### 2.1.3 Wireless Energy Transmission

Electrical stimulation therapy devices rely on wireless energy transmission technology as a continuous and stable power source.^[^ ^14^ ^]^ There are three main principles of radio energy transmission, namely electromagnetic induction, electron magnetic resonance, and electromagnetic radiation. Electromagnetic induction, which involves placing two coils in close proximity, is the most common type of wireless transmission. When an electric current flows through one coil,^[^ ^15^ ^]^ the resulting magnetic flux acts as a medium, causing an induced electromotive force in the other coil. As a technology that can implement energy delivery without relying on any physical connection, wireless energy transmission has excellent convenience, mobility, and flexibility, and its corresponding equipment has been used in skin repair,^[^ ^16^ ^]^ neuromuscular repair, and other fields.^[^ ^17^ ^]^ However, this technology has several disadvantages: (1) Due to its reliance on electromagnetic waves for energy transmission, this technology is highly susceptible to external interference; (2) an increase in distance leads to a decrease in the efficiency of electrical energy transmission; and (3) it necessitates additional power sources for the device's operation, resulting in a higher energy consumption compared to wired devices.^[^ ^15^ ^]^

#### 2.1.4 Self‐Powered Technology

Self‐powered technology is a new energy supply method that does not depend on external energy supplies. The main self‐powered technologies for tissue engineering are nanogenerators (NGs), biofuel cells, and photovoltaic cells.

#### 2.1.5 Piezoelectric Nanogenerator (PENG)

PENG is an NGs designed based on the piezoelectric effect that can collect tiny mechanical energy at the nanoscale and convert it into electricity. The piezoelectric effect is a phenomenon in which a material generates an internal electric potential under the stress.^[^ ^17^ ^]^ Taking zinc oxide (ZnO) as an example, Zn^2+^ and O^2−^are tetrahedral distributions, and their positive and negative charge centers overlap. External mechanical forces along the C‐axis shift the positive and negative charge centers to form a dipole moment, and the superposition of dipole moments of each crystal unit on the macroscopic surface forms a “piezoelectric potential”. When the piezoelectric material is connected to an external circuit, the piezoelectric potential drives electrons through the circuit, establishing a new equilibrium state. Materials such as ZnO, zinc stannate (ZnSnO_3_), barium titanate (BaTiO_3_), lead zirconate titanate (PZT), polyvinylidene fluoride (PVDF), and its copolymer P(VDF‐TrFE) can serve as piezoelectric materials. The PENG's advantages, including low power consumption, simple design, excellent flexibility, and mechanical stability, make it a valuable source for electrical stimulation therapy.^[^ ^18^ ^]^

#### 2.1.6 Triboelectric Nanogenerator (TENG)

The TENG, or triboelectric nanogenerator, operates based on the triboelectric effect and electrostatic induction.^[^ ^19^ ^]^ It is typically composed of friction layer, electrode layer, and external load. When two friction layers make contact, they acquire equal but opposite charges. Upon separation, these charges generate an induced potential difference on the electrode behind the material. When connected to an external load, the induced potential difference drives the charge flow to balance the potential difference and form a current. TENGs operate in four different modes: vertical contact‐separation, lateral sliding, single‐electrode, and freestanding triboelectric‐layer.^[^ ^19^ ^]^ The TENG has the characteristics of self‐powered (generating electricity through human movement without additional power supply), controllable parameters, lightweight and portable, flexible, wearable, low cost, and high safety (high voltage and low current). The development of self‐powered electrical stimulation devices using TENGs provides a promising method for electrical stimulation therapy.

#### 2.1.7 Biofuel Cells

A biofuel cell is a unique type of fuel cell that uses organic matter as fuel and employs enzymes as catalysts, either directly or indirectly. Glucose is the typical biofuel cell fuel, and oxygen is the oxidant needed for chemical reactions to occur. Research has demonstrated that biofuel cells can also utilize lactic acid and vitamin C as fuel.^[^ ^20^ ^]^ Microorganisms, organelles, and enzymes are the three main types of catalysts used in biofuel cells, with some non‐enzymatic proteins also serving as catalysts. The organism widely distributes these substances throughout its body. Furthermore, biofuel cells exhibit mild reaction conditions (normal temperature, normal pressure, and neutrality) and good biocompatibility, as the body provides the fuel, catalyst, and oxidant.^[^ ^21^ ^]^ Therefore, biofuel cells can directly convert organic matter in the body into electricity, as a power supply for electrical stimulation equipment.

#### 2.1.8 Photovoltaic Cells

Photovoltaic cells are devices that harness the photovoltaic effect of semiconductors to convert light energy into electrical energy.^[^ ^22^ ^]^ When light of specific wavelengths strikes P‐N junctions, which are created by combining P‐type and N‐type semiconductors, it generates electron‐hole pairs in the junction region. The electric field in the P‐N junction causes the holes to move from the N‐type region to the P‐type region, while the electrons shift from the p region to the n region, leading to the formation of the current upon connecting the external load. With the development of semiconductor materials, researchers have gradually developed photovoltaic cells based on monocrystalline silicon, polysilicon, perovskite, lead‐free perovskite, and other types.^[^ ^22^ ^]^ Due to their good biocompatibility, permanence, flexibility, and cleanliness, silicon‐based photovoltaic cells are suitable for direct use in electrical stimulation therapy or as a power source for electrical stimulation therapy equipment.^[^ ^23^ ^]^

### 2.2 Electrode Materials

Applying electrical stimulation to tissue necessitates the use of appropriate electrodes, in addition to the power supply of the electrical stimulation device. Electrodes need to have good electron or ion transport properties, biocompatibility, an appropriate modulus, and flexibility to fit into tissues. The materials selected for the electrode are mainly in the following categories: metal materials, conductive polymers, semiconductor materials, and inorganic nanomaterials.

#### 2.2.1 Metal Materials

Various electronic devices widely use metal materials as electrodes due to their high electrical conductivity. Metal materials used as electrodes for electrical stimulation therapy are usually synthesized by physical vapor deposition, electrochemical deposition, or sputtering techniques to ensure their high conductivity. Moreover, electrodes need to be biocompatible with tissues. The metal electrodes commonly used for electrical stimulation therapy are mainly gold (Au), platinum (Pt), argentum (Ag), and platinum‐iridium alloys.^[^ ^5^ ^]^ Because these materials have excellent electrochemical stability and wear resistance, biological fluids cannot destroy them, allowing them to exist in the human body for a long time.^[^ ^24^ ^]^ This type of non‐degradable metal electrode is suitable for long‐term electrical stimulation therapy, such as pacemakers and neurostimulators, which need to provide electrical stimulation stably for a long time and are not decomposed and absorbed by the body.^[^ ^25^, ^26^ ^]^ For temporary electrical stimulation therapy, such as bone and nerve repair,^[^ ^27^, ^28^ ^]^ in order to avoid secondary damage when the implanted electrode is removed, degradable metals such as iron (Fe), magnesium (Mg) and molybdenum (Mo) are undoubtedly suitable electrode materials.^[^ ^29^ ^]^

#### 2.2.2 Conductive Polymer

Conductive polymers are mainly divided into two categories: composite conductive polymer materials and intrinsic conductive polymer materials.^[^ ^30^ ^]^ Composite conductive polymer materials are usually synthesized by embedding conductive fillers (such as carbon nanomaterials, metal nanoparticles, ionic solutions, etc.) into a polymer matrix using chemical polymerization, solution mixing, or electrospinning.^[^ ^31^ ^]^ By selecting different polymers, conductive materials, and water content (mainly conductive hydrogels), the conductivity, modulus, and flexibility of the material can be adjusted. Intrinsic conductive polymer materials are usually synthesized by chemical polymerization or electrochemical polymerization, such as polypyrrole, poly(3,4‐ethylenedioxythiophene) (PEDOT), etc.^[^ ^32^ ^]^ The molecular structure of this type of material is a conjugated long chain, and the delocalized electrons in the double bonds can migrate along the polymer chain to promote the passage of current. It has a good match with the resistance value of biological tissues and can reduce the signal loss between electrodes and cells. Conductive polymer materials generally have good biocompatibility, flexibility, and adjustable conductivity.^[^ ^33^ ^]^ They can adapt to the complex human environment and meet the needs of different tissue repairs, which are often used as electrode materials for nerve regeneration,^[^ ^34^ ^]^ muscle repair,^[^ ^35^ ^]^ and wound repair.^[^ ^36^ ^]^

#### 2.2.3 Semiconductor Materials

Semiconductor materials can be used as electrical stimulation electrodes, mainly because their conductivity is between that of conductors and insulators, the conductivity can be changed by regulating the doping level, and they have good biocompatibility and mechanical properties.^[^ ^37^ ^]^ These electrodes are usually synthesized by chemical vapor deposition, physical vapor deposition, or solution processing. When the semiconductor comes into contact with the electrolyte, it distributes its residual charge on the surface of the electrode, forming a space charge layer similar to the ionic double layer in the solution.^[^ ^38^ ^]^ Silicon has good biocompatibility, mechanical stability, electrical properties, and integrability, and is the most commonly used material for semiconductor electrodes. In order to improve the photoelectric performance of silicon electrodes, the silicon electrodes are usually modified with Au nanoparticles to enhance the efficiency of carrier transport and light absorption.^[^ ^39^ ^]^ Photoelectric technology typically combines semiconductor electrodes for electrical stimulation of the nervous system.^[^ ^23^ ^]^ P‐type silicon or N‐type silicon can also directly use its hole and electron abundance characteristics for direct neural regulation.^[^ ^24^ ^]^

#### 2.2.4 Carbon Nanomaterials

Carbon‐based materials, such as carbon nanotubes and graphene, are usually prepared by chemical vapor deposition (CVD), ultrasonic mechanical exfoliation, pyrolysis, and other methods.^[^ ^40^ ^]^ Plants, petroleum, and other sources provide a wide range of carbon‐based materials. Carbon nanomaterials are usually prepared into electrical stimulation electrodes using direct coating, transfer printing, solution processing, and other technologies, which can achieve uniform deposition and patterning of carbon nanomaterials on flexible substrates.^[^ ^5^ ^]^ Electrodes based on carbon‐based nanomaterials have extremely low impedance and high sensitivity, which can provide higher signal quality; they have single ingredient and excellent biocompatibility, and can reduce immunogenicity and inflammatory response; electrodes of different shapes and hardness can be mixed with flexible substrate materials to adapt to different types of tissues.^[^ ^41^ ^]^ Therefore, carbon‐based electrodes are widely used in nerve and muscle repair,^[^ ^42^ ^]^ and can effectively apply electrical stimulation to tissues to promote cell proliferation and tissue regeneration.

## 3 Regulation of Cellular Activity by Electric Field

**Figure 2:**
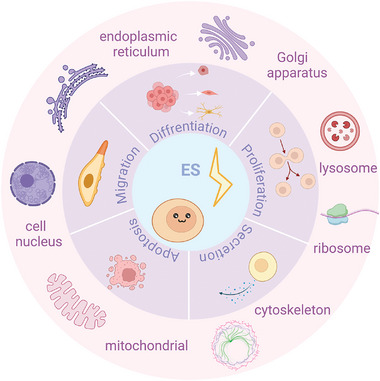
Electrical stimulation influences cellular behavior through key organelles and molecules.

Bioelectrical activity is one of the fundamental factors in maintaining life activities. Within the body, electric fields are instrumental in regulating vital cells across various tissues and organs. This endogenous bioelectrical activity is essential for regulating cell growth, differentiation, migration, apoptosis, and various other physiological processes.^[^ ^5^ ^]^ Research has shown that membrane potential and electric fields profoundly affect cellular behaviors, such as polarity, adhesion, migratory speed, and signal transduction.^[^ ^43^, ^44^, ^45^ ^]^ On this basis, electrical stimulation therapy, as an innovative treatment approach, applies external electric fields to mimic and enhance the effects of endogenous electric fields, modulating the electrophysiological activity of cells. This, in turn, influences cellular behavior, providing a new pathway for tissue repair and regeneration. (**Figure** **2**)

### 3.1 Cell Migration

Cell migration involves collective polarization, mechanical coupling, and cytoskeletal dynamics, and is the result of the coordination of multiple factors.^[^ ^46^, ^47^ ^]^ Electrical stimulation influences cell migration through multiple pathways—including cytoskeletal assembly, cytoskeletal assembly, Golgi apparatus function, calcium channel activation, and membrane potential regulation‐and enhances the migration of various cell types, such as fibroblasts, endothelial cells, osteoblasts, and neural progenitor cells, thereby playing a critical role in tissue repair and regeneration.^[^ ^48^, ^49^, ^50^, ^51^ ^]^ For example, cells extend and form a leading edge toward the signal direction in response to directional cues within wounds. To migrate effectively, the Golgi apparatus regulates the formation of the cell leading edge through directional secretion. Activation of primary polarization signals at the cell's front end relays these signals to the Golgi apparatus, initiating a second set of signaling events that control subsequent reorganization of the secretory pathway. The Golgi apparatus localization signaling pathway is important for polarizing cells in clusters migrating around the Microtubule Organizing Center (MTOC), giving migrating cells polarity that aligns with those at the wound edge.^[^ ^52^, ^53^ ^]^ In addition, electric fields can activate voltage‐gated Ca^2+^ or Na^+^ ion channels, triggering cellular ion influx and downstream signaling via ion transporters such as Na⁺/K⁺‐ATPase, sodium‐hydrogen exchanger 3 (NHE3), and cytoskeletal polarization.^[^ ^54^, ^55^ ^]^ Membrane receptors associated with cell migration, such as epidermal growth factor receptor (EGFR), acetylcholine receptor (AchR), and integrins, can also respond to electric fields and activate downstream PI3K/Akt and MAP/ERK signaling pathways.^[^ ^54^, ^56^ ^]^ In summary, electrotaxis supports self‐regeneration and wound healing. These intrinsic tissue effects can be harnessed and enhanced clinically through external electrical stimulation, thereby accelerating the healing process.^[^ ^55^, ^57^ ^]^

### 3.2 Cell Proliferation

Cell proliferation is a fundamental physiological process in cell biology, encompassing key stages like cell cycle regulation and mitosis. Cell cycle regulation is a critical process, and electric fields have an impact on all aspects of the regulation of cell cycle proteins, including gene transcription, translation, and synthesis.^[^ ^58^ ^]^ First, electric fields can affect the transcription process in the cell nucleus. According to research, electric fields with different parameters can change gene expression through the nuclear factor κB (NF‐κB) pathway,^[^ ^59^, ^60^ ^]^ which can speed up or slow down the process of cell proliferation. Second, electric fields can also affect the translation process. Translation is the process of translating mRNA into protein, and one of the crucial organelles is the ribosome. Electric field stimulation may affect the rate of ribosome‐mediated protein synthesis by regulating the activity of metabolic enzymes, thereby influencing the level of protein synthesis.^[^ ^61^ ^]^ Through their effects on transcription and translation, electric fields can control the quantity and types of proteins synthesized by cells, thereby impacting cell proliferation. This may also be related to the fact that electric fields may increase the exchange of nutrients in the cell.^[^ ^62^ ^]^ In addition, electrophoretic mobility may lead to the accumulation of molecules on the cell membrane surface, causing conformational changes in protein molecules and asymmetric redistribution of proteins within the membrane (changes in cell symmetry), ultimately affecting cell proliferation.^[^ ^63^, ^64^ ^]^ Typically, cells with very high resting potentials (such as muscle cells and neurons) exhibit lower mitotic activity; electric fields that alter cell transmembrane potential may lead to enhanced cell proliferation.^[^ ^64^, ^65^ ^]^ Researchers have demonstrated that electric fields can stimulate the proliferation of various cell types, including fibroblasts, human umbilical vein endothelial cells (HUVECs), myocytes, mesenchymal stem cells, and hair follicle cells.^[^ ^66^, ^67^, ^68^, ^69^ ^]^

### 3.3 Cell Differentiation

Under exogenous electrical stimulation, cell differentiation is regulated through the combined effects of multiple signal transduction pathways, cytoskeletal reorganization and actin distribution, surface receptor redistribution, ion channel activity, and membrane potential modulation.^[^ ^70^ ^]^ Studies have demonstrated that electric fields can direct the targeted differentiation of various cell types, including guiding mesenchymal stem cells to differentiate into neural stem cells and osteoblasts.^[^ ^71^, ^72^, ^73^, ^74^ ^]^ Exogenous electric fields induce cell membrane depolarization, activating voltage‐gated Ca^2+^ channels and leading to an increase in intracellular calcium concentration,^[^ ^75^ ^]^ and the depolarization of the cell membrane opens voltage‐dependent calcium channels in pluripotent embryonic cells.^[^ ^76^ ^]^ Electric fields regulate calcium storage in the endoplasmic reticulum through Ca^2+^ channels.^[^ ^77^, ^78^, ^79^ ^]^ For instance, electric field‐induced changes in endoplasmic reticulum (ER) calcium levels can influence the calcium/calmodulin‐dependent protein kinase II (CaMKII) pathway. Due to actin reorganization, electrical stimulation may also affect tension within the cytoskeleton.^[^ ^80^ ^]^ In this way, the cytoskeleton and cytoskeleton‐associated processes may be altered,^[^ ^81^ ^]^ one of which is the electric field‐induced redistribution of membrane receptors.^[^ ^82^ ^]^ Additionally, electric fields can alter communication between the ER and mitochondria, a vital interaction for maintaining cellular bioenergetics and redox signaling during differentiation.^[^ ^77^ ^]^ ER‐mitochondria crosstalk, influenced by electric fields, can alter mitochondrial function, reactive oxygen species (ROS) production, and cellular energy utilization.^[^ ^83^, ^84^, ^85^ ^]^ All of these are important for deciding a cell's fate and the process of differentiation.

### 3.4 Cell Apoptosis

Studies have demonstrated that electric fields impact multiple aspects of cell apoptosis, thereby affecting tissue repair.^[^ ^86^ ^]^ The regulation of apoptosis is important for maintaining cellular homeostasis within tissues and for eliminating abnormal cells.^[^ ^87^ ^]^ Electrical fields can activate pro‐apoptotic factors by altering cell membrane permeability, such as B‐cell lymphoma 2(Bcl‐2) family proteins, which play a critical role in the intrinsic pathway of apoptosis. The Bcl‐2 family includes pro‐apoptotic proteins (such as Bcl‐2‐associated X protein and Bcl‐2 antagonist/killer) and anti‐apoptotic proteins (such as Bcl‐2 and Bcl‐xL), with their balance directly influencing cell survival or death.^[^ ^58^, ^88^ ^]^ Additionally, electric fields can influence apoptosis decisions by regulating the redistribution of death receptor signaling pathways (such as the Fas/Fas ligand pathway) on the cell membrane. These signaling pathways are particularly crucial for controlling the apoptosis of inflammatory cells.^[^ ^89^, ^90^, ^91^, ^92^ ^]^ Studies have shown that electric fields can regulate the expression of these proteins, thereby modulating the sensitivity of cell apoptosis.^[^ ^58^ ^]^ In the early stages of tissue repair, electric fields promote apoptosis in inflammatory cells by targeting the Bcl‐2 family and death receptor pathways. This helps mitigate excessive inflammatory responses, aiding in the clearance of necrotic tissue and creating a favorable environment for wound healing. A moderate inflammatory response supports the normal progression of healing, while excessive inflammation can delay healing or even lead to chronic inflammation.^[^ ^93^, ^94^ ^]^ Electrical stimulation effectively reduces unnecessary inflammatory responses by inducing apoptosis in inflammatory cells, facilitating a smooth transition of the healing process into the tissue remodeling phase. As the wound enters the remodeling stage, the number and function of fibroblasts become critical factors in determining repair quality. Electric fields regulate the directional migration and apoptosis of fibroblasts, preventing excessive scarring from an overabundance of fibroblasts while also avoiding incomplete healing due to an insufficient number of fibroblasts.^[^ ^95^ ^]^ By modulating the cytoskeleton and signaling pathways (such as the PI3K/Akt pathway), electrical stimulation promotes the directional migration of fibroblasts and the synthesis of their extracellular matrix, thereby optimizing tissue repair and reducing scar formation.^[^ ^96^, ^97^, ^98^ ^]^ Beyond wound healing, electric fields play an important role in bone healing. Apoptosis in osteoclasts and osteoblasts is vital for maintaining bone homeostasis and function. Excessive activity of osteoclasts can lead to increased bone resorption, disrupting bone balance. Similarly, in bone healing, electric fields regulate the apoptosis of osteoclasts and osteoblasts to maintain bone homeostasis, preventing excessive bone resorption and abnormal bone density.

### 3.5 Cell Secretion

The process of cell secretion under the influence of electric fields involves complex interactions among organelles and molecules. Research demonstrates that intracellular ATP supports the reorganization of the actin cytoskeleton, and electrical stimulation can indirectly boost ATP production by guiding proton migration toward the mitochondrial membrane. As a result, electrical stimulation indirectly affects cell secretion.^[^ ^99^, ^100^, ^101^, ^102^ ^]^ Furthermore, activation of transduction pathways can induce stress responses in the endoplasmic reticulum (ER), the primary site for protein synthesis and modification within cells.^[^ ^77^ ^]^ This can change the folding routes for proteins and affect their structure. The Golgi apparatus is responsible for the later stages of protein modification.^[^ ^103^ ^]^ Electric field stimulation changes the structure and function of the Golgi apparatus, affecting its role in protein processing and packaging. Additionally, electric fields can regulate protein transport by modulating intracellular calcium levels, thereby influencing cell secretion dynamics. This modulation impacts the movement and sorting of secretory vesicles within the cell, as well as the overall secretion process. Key molecules in tissue repair, such as vascular endothelial growth factor (VEGF), fibroblast growth factor (FGF), and insulin‐like growth factor (IGF), play essential roles in angiogenesis, granulation tissue formation, and other repair processes.^[^ ^104^, ^105^, ^106^, ^107^, ^108^ ^]^ Researches indicate that electric fields can enhance the secretion of molecules mentioned above by various cell types (mesenchymal stem cells, fibroblasts, etc.).^[^ ^108^ ^]^

## 4 Applications of Electrical Stimulation Therapy in the Regulation of Tissues Related to Plastic Surgery

Tissue transplantation is a widely employed and effective technique that facilitates the regeneration and repair of damaged tissues, including skin, its appendages, bones, cartilage, and tendons.^[^ ^109^ ^]^ Following transplantation, electrical stimulation therapy promotes the growth and healing of new tissues while reducing postoperative pain and muscle atrophy. By appropriately setting electrical stimulation parameters, this therapy regulates the proliferation and differentiation of tissue cells, thereby accelerating the healing process and enhancing the survival rate and functional recovery of transplanted tissues.^[^ ^110^, ^111^ ^]^ To further support tissue repair, the restoration of blood vessels and nerves is essential.^[^ ^112^ ^]^ The circulatory system ensures that injured tissues receive adequate oxygen and nutrients, promoting tissue regeneration and restoration. In clinical practice, electrical stimulation therapy can be combined with microvascular surgery and nerve regeneration techniques to develop comprehensive treatment plans.^[^ ^4^, ^113^ ^]^ This combination enhances angiogenesis and nerve function, resulting in improved healing outcomes for injured tissues. Consequently, patients can regain both aesthetic appeal and functional abilities, significantly enhancing their overall quality of life.

In summary, plastic surgery continually explores new treatment modalities for tissue repair. Electrical stimulation therapy serves as an effective adjunctive approach, offering patients more comprehensive treatment options. Through the optimized application of electrical stimulation protocols, plastic surgeons can achieve better repair and reconstruction of damaged tissues, ultimately leading to improved surgical outcomes and enhanced recovery experiences. (**Table** **1**).

**Table 1:** The summary of electrical stimulation repair techniques for orthopedic surgery.

Tissue	Device	Electrical output	Animal or cell model	Treatment effect	Ref.
Skin	TENG	Voltage: 0.2 V‐2.2 V Frequency: 110 Hz min^−1^ Time: 24 h day^−1^, 3 days	Rats, fibroblasts	Promoted granulation tissue formation	[114]
	TENG	Voltage: 25 V Current: 1 µA Frequency: 1 Hz Time: 24 h day^−1^, 12 days	Mice, fibroblasts	Promoted granulation tissue formation	[115]
	Biofuel cells	Voltage: 0.04 V‐ 0.94 V Time: 24 h day^−1^, 7 days	Mice, Caco‐2	Promoted re‐epithelialization, reduce scarring	[10]
	TENG	Voltage: 20 V Current:1 µA Frequency: 2 Hz Time: 24 h day^−1^, 12 days	Mice, HaCaT	Promoted re‐epithelialization, vascularization, HF formation	[24]
	PENG	Voltage: 0 – 30 V Frequency: 1 Hz Time: 24 h day^−1^, 4 days	Rats, fibroblasts	Promoted granulation tissue formation and re‐epithelialization	[116]
	TENG	Voltage: 1.44 V Current: 100 nA Time: 24 h day^−1^, 7 days	Pigs, HaCaT, THP‐1	Promoted granulation tissue formation and re‐epithelialization, reduce scarring	[117]
	Wireless energy transmission	Voltage: 0–4.3 kV mm^−1^ Time: 24 h day^−1^, 8 days	Rats, fibroblasts	Promoted re‐epithelialization, vascularization and granulation tissue formation	[118]
	Wireless energy transmission	Voltage: 0 – 2 V Frequency: 13.56 MHz Time: 6 h day^−1^, 9 days	Mice	Promoted granulation tissue formation and re‐epithelialization	[14]
	TENG	Voltage:0 ‐300 mV Time: 24 h day^−1^, 12 days	Rats	Regulated macrophage polarization	[119]
Vessel	PENG	Voltage: 322.04 ± 12.85 mV Time: 24 h day^−1^, 5 days	CAM, HUVECs	Promoted vascularization	[120]
Follicle	TENG	Voltage: 0 – 0.2 V Time: 24 h day^−1^, 18 days	Rats, nude mice 3T3	Promoted HF formation	[121]
Nerve	Galvanic cells	Voltage: 3 mV Frequency:1 Hz Time: 24 h day^−1^, 12 weeks	Rats, DRG neurons	Promoted axonal remyelination	[122]
	PENG	Voltage: 0 – 17.9 V Current: 0 – 2.6 µA Frequency: 10 Hz Time: 20 min per 2 days, 4 weeks	Rats	Promoted endogenous neurogenesis and angiogenesis	[123]
	Wireless energy transmission	Voltage: 0 ‐1.2 V Current: 0 – 640 µA Frequency: 5 Hz Time: 20 min per 2 days, 4 weeks	Rats, neural stem cell	Promoted remyelination, accelerate axon regeneration, facilitate endogenous neural stem cell differentiation	[124]
	Wireless energy transmission	Voltage: 0 – 40 mV Current: 0 – 40 nA Frequency: 10 Hz Time: 1 h day^−1^, 8 weeks	Mice	Activated nerves and promote regeneration	[125]
Bone	Optoelectronic scaffold	Voltage: 0‐ 180 mV Current: 0–6 nA Frequency: 0.2 Hz Time: 20 min per 2 days, 20 days	Rats, hBMSCs	Promoted cell proliferation and osteogenic differentiation	[23]
	Galvanic cells	Current:0‐4.5 µA Time: 24 h day^−1^, 12 weeks	Rats, BMSCs	Upregulated calcium signaling pathway, promote osteogenic differentiation	[126]
	PENG	Voltage: 0‐ 220 mV Time: 24 h day^−1^, 10 days	Rats, MSCs	Recruited stem cells, promoted osteogenic differentiation	[18]
	PENG+TENG	Voltage:0‐0.6 V Time: 24 h day^−1^, 14 days	Rats, BMSCs	Promoted the proliferation, migration, and differentiation of BMSC	[127]
Cartilage	PENG	Voltage: 0–3.6 V Frequency: 1 Hz Time: 20 min day^−1^, 2 months	Rabbits, ADSCs	Promoted cell migration, upregulate calcium signaling pathway	[128]
	PENG	Voltage: 0–9.3 V Time: 18 weeks	Rabbits	Upregulated calcium signaling pathway	[129]
Tendon	PENG	Voltage: 0–3 V Time: 24 h day^−1^, 14 days	Rats, rBMSCs	Induce directional differentiation	[130]
	semiconductor floating‐gate memory circuit	Current: 0.04−0.6 mA Frequency: 10 Hz Time: 15 mins	Mice, PC‐12	Reduced nerve damage, lower inflammatory cytokines	[131]

TENG, triboelectric nanogenerator; PENG, piezoelectric nanogenerator; HUVECs, human umbilical vein endothelial cells; hBMSCs, human bone mesenchymal stem cells; ADSCs, Adipose Derived Stem Cells.

### 4.1 Wound Healing

#### 4.1.1 Skin

The skin, as the largest organ of the human body, serves as a vital protective function. However, various factors such as wounds, surgical interventions, or diseases can compromise the skin, leading to chronic wounds or excessive scars that can cause significant physical and psychological distress to patients.^[^ ^132^, ^133^ ^]^ Therefore, wound repair and scar prevention have become central research focuses in the field of plastic surgery. Wound healing encompasses four interconnected stages: (1) Hemostasis, (2) Inflammation, (3) Proliferation, and (4) Remodeling.^[^ ^29^ ^]^ These stages are not independent, but rather intertwined, involving the collaborative participation of various inflammatory cells, repair cells, the extracellular matrix, and other components. The management of chronic refractory wounds remains a significant challenge in plastic surgery. Electrical stimulation therapy is instrumental in the repair of skin wounds and the prevention of scars, and its effects permeate the entire wound healing process.^[^ ^48^, ^134^, ^135^ ^]^

**Figure 3:**
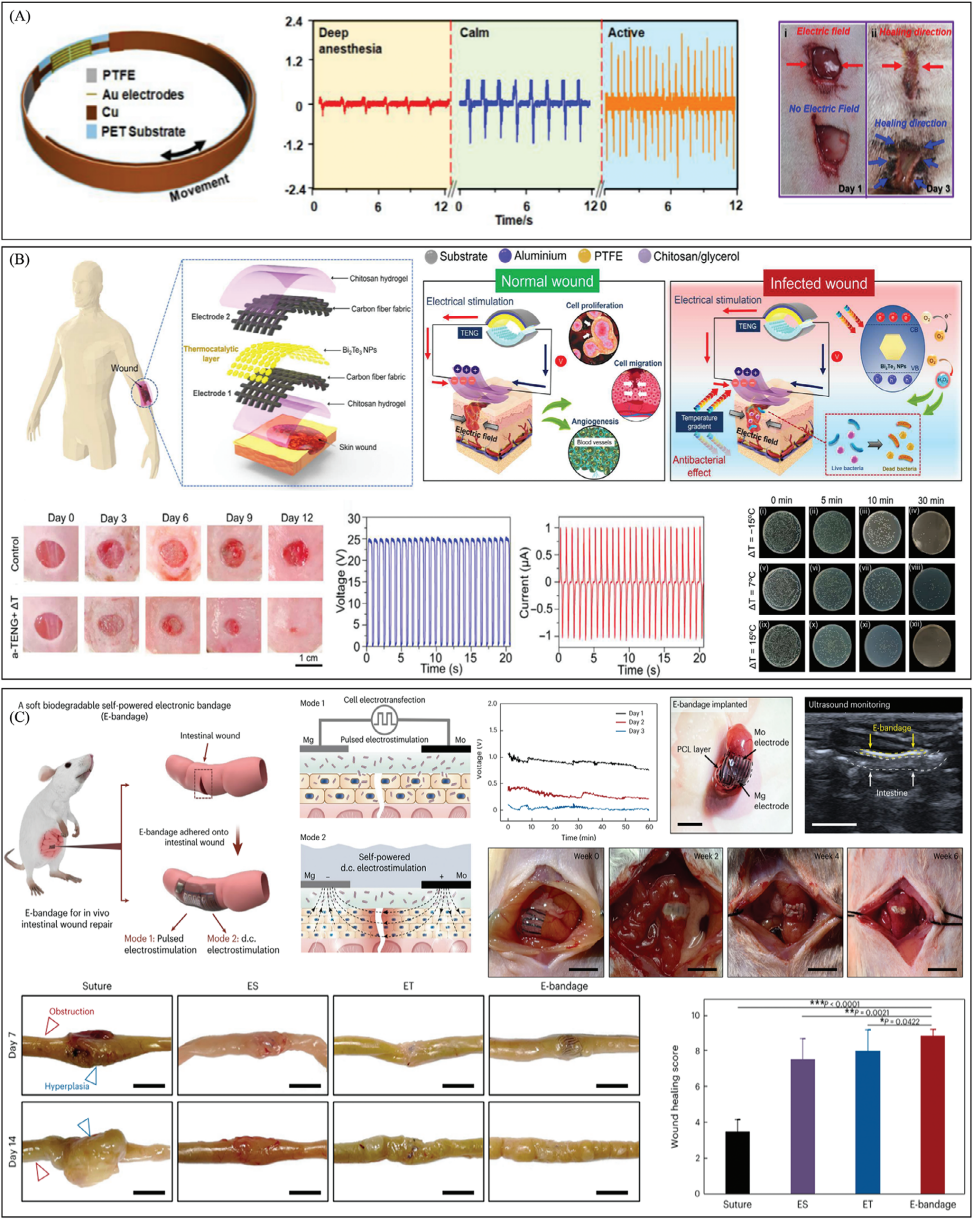
The development of electrical stimulation therapy in the field of wound healing. A) An efficient electrical bandage promotes the migration of epidermal cells by offering a continuous electrical stimulation driven by respiratory movement, thereby promoting wound repair. Reproduced with permission.^[^ ^114^ ^]^ Copyright 2018, Publisher, American Chemical Society. B) A carbon fiber fabric electrode triggered by temperature differences, which could promote the formation of granulation tissue and inhibit bacterial growth at the wound site to promote skin repair. Reproduced with permission.^[^ ^115^ ^]^ Copyright 2023, Publisher, American Association for the Advancement of Science (AAAS). C) A flexible degradable bandage powered by a primary battery promotes wound repair by reducing scar tissue proliferation. Reproduced with permission.^[^ ^10^ ^]^ Copyright 2024, Publisher, Springer Nature.

The epidermis, as the outermost layer of the skin, primarily consists of epithelial cells. The repair process is intricately linked to the rate of wound healing, where the basal skin potential mechanism significantly influences epithelial cell migration.^[^ ^136^, ^137^ ^]^ The primary findings of the study suggest that a significant factor in accelerating wound healing through electrical stimulation is the electric field's ability to provide directional signals for the migration of epithelial cells. Anodic direct current stimulation can enhance the recovery rate of endogenous electric fields, thereby guiding epithelial cells to expedite wound healing.^[^ ^56^, ^138^ ^]^ In contrast to traditional electrical stimulation devices, such as DC power supplies (outputting DC) and signal generators (outputting AC and PC), current electrical stimulation therapy methods have essentially achieved the miniaturization and portability of electrical stimulation devices. Long et al. designed an efficient electrical bandage that could offer continuous electrical stimulation driven by breath.^[^ ^114^ ^]^ (**Figure** **3**A) In this paper, they used an electric field strength of ≈250 V m^−1^ and reached the highest peak‐to‐peak voltage amplitude (V_pp_) of 2.2 V at a rate of 110 per minute. Three days later, the wounds exposed to the electric field showed improved healing, whereas the control wounds remained open and showed disordered healing. This phenomenon confirmed that NG's small, discrete electric field can guide and promote wound healing in its direction. Granulation tissue primarily consisted of fibroblasts and the collagen they produce. During the initial stages of wound healing, an elevation in collagen levels aids in the proper healing process. Numerous studies have demonstrated that electrical stimulation significantly enhances fibroblast proliferation and collagen secretion, thereby promoting granulation tissue formation. Thermally catalyzed bismuth telluride nanosheets (Bi_2_Te_3_ NPs), functionalized on a carbon fiber fabric electrode, form the basis of self‐powered multifunctional dressing. Temperature differences trigger this dressing, which controllably generates hydrogen peroxide to effectively inhibit bacterial growth at the wound site.^[^ ^115^ ^]^ (Figure 3B) A wearable TENG is connected to an integrated electrode, producing a DC electric field capable of penetrating the dermis and amplifying the endogenous electric field. In the electrical stimulation study, the TENG provided an output voltage of 25 V at 1 Hz, facilitating the proliferation and migration of fibroblasts. Additionally, thermally catalyzed Bi_2_Te_3_ nanoparticles (NPs) within the electroactive layer functionalized the composite wound dressing, exhibiting significant antibacterial efficacy in vitro. Therefore, we can further use the wearable, self‐powered dressing as an in‐situ platform for treating bacterially infected wounds. In addition to skin wounds, intestinal wounds could also be performed since they are both epithelial tissues. Wu et al. showed a soft and biodegradable electronic bandage that can promote tissue repairment and reduce abnormal hyperplasia.^[^ ^10^ ^]^ (Figure 3C) This device used two types of electrical stimulation to help wounds heal: pulsed electrical stimulation causes epithelial cell electrotransfection (ET), which increased the production of healing factors like epithelial growth factor; and direct current electrical stimulation from biodegradable Mg and Mo microelectrodes in galvanic cells could increase the release of healing factors from transfected cells. The output of the digital microelectrode pair was a DC electric field that lasted for 3 days. This bandage adheres firmly to the tissue. In a mouse model of small intestinal wound healing, this device effectively promoted healing and reduced the occurrence of intestinal obstruction. Following a period of 4 weeks since implantation, the device had completely dissolved. Compared with other electrical stimulation devices, the degradability of this device makes it have a broader application prospect in future plastic surgery operations.

**Figure 4:**
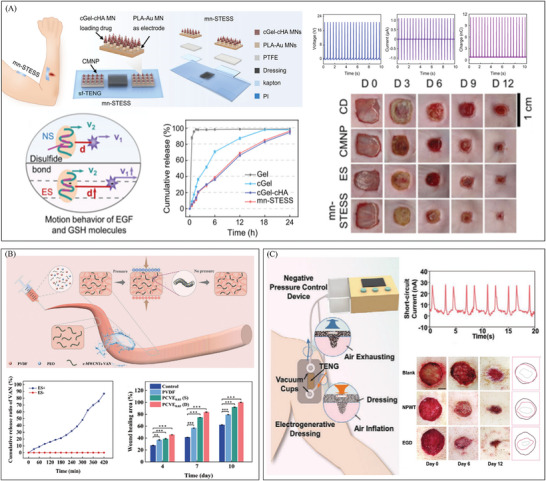
Clinical application of combined treatment with electrical stimulation in wound repair. A) A microneedle‐based self‐powered transcutaneous electrical stimulation system (mn‐STESS) uses electrical stimulation as an adjuvant to increase EGF levels then promote wound healing. Reproduced with permission.^[^ ^24^ ^]^ Copyright 2022, Publisher, Springer Nature. B) A self‐powered wound dressing that combines an electric field ‐driven drug release mechanism with electrical stimulation therapy. Reproduced with permission.^[^ ^116^ ^]^ Copyright 2024, Publisher, WILEY‐VCH. C) An electrogenerative dressing (EGD) to achieve the combined therapy using electrical stimulation and negative‐pressure wound treatment (NPWT). Reproduced with permission.^[^ ^117^ ^]^ Copyright 2023, Publisher, WILEY‐VCH.

In addition, electrical stimulation can directly facilitate tissue repair and may also be integrated with other therapeutic modalities to enhance wound healing. Endogenous sustained‐release technology is commonly designed based on average pharmacokinetic parameters observed in healthy individuals. However, a single drug formulation often fails to account for individual variability in pharmacokinetics under varying pathological conditions, thereby hindering the achievement of optimal therapeutic outcomes.^[^ ^139^, ^140^, ^141^ ^]^ Thus, Yang et al. described mn‐STESS, a self‐powered transcutaneous electrical stimulation system based on microneedles that improves the pharmacodynamics of EGF to help wounds heal.^[^ ^24^ ^]^ (**Figure** **4**A) This system consisted of two‐stage composite microneedle patches (CMNPs) and a sliding free‐standing TENG (sf‐TENG), using electrical stimulation serving as an electrical adjuvant to enhance the pharmacodynamics of epidermal growth factor (EGF) B‐cell lymphoma 2 family proteins. When the finger slid at a frequency of 2 Hz, an electrical output sufficient for wound repair was generated with an open circuit voltage (V_OC_) of 20 V and a short circuit current (I_SC_) of 1 µA. By increasing the penetration of EGF, mn‐STESS improved pharmacodynamics without affecting the drug release rate. In the rat back wound model, mn‐STESS showed better wound healing performance than electrical stimulation and CMNP alone, with a healing rate 18% higher than CMNP and 36% higher than electrical stimulation in the first 6 days.

Drug delivery systems can also combine with electrical stimulation as a treatment to promote wound healing. Sun et al. presented a self‐powered wound dressing that could control the “lock on/off” of drug release via electrical stimulation.^[^ ^116^ ^]^ (Figure 4B) The system was composed of polyvinylidene fluoride (PVDF) and carboxylated carbon nanotube multi‐walled composite with vancomycin hydrochloride (c‐MWCNTs‐VAN), where PVDF acted as a energy‐harvesting system to power the entire system to achieve the dual function of electrical stimulation. Electrical stimulation could autonomously regulate drug release and effectively alleviate drug resistance. Moreover, as a bioelectric stimulation signal, electrical stimulation accelerated wound healing by modulating vascular regeneration, collagen deposition, epithelialization, and the expression of related growth factors. The integration of an all‐smart electric field‐responsive drug release switch with electrical stimulation treatment markedly improved wound healing compared to the PVDF‐onlyelectrical stimulation treatment group.

Besides being combined with chemotherapy, electrical stimulation therapy can also be used in combination with physical therapy. Luo et al. developed an electrogenerative dressing (EGD) to achieve the combined therapy using electrical stimulation and negative‐pressure wound treatment (NPWT).^[^ ^117^ ^]^ (Figure 4C) The system comprised two core components: NPWT device with foam dressing, and a self‐powered electrical stimulation system, which included a TENG, rectifier, and electrodes. In recent years, NPWT has clearly emerged as a superior approach for the treatment of chronic wounds.^[^ ^142^, ^143^ ^]^ However, NPWT could not only remove harmful exudates, but also lead to electrolyte loss in the wound, which in turn affects the wound edge electric field and delays wound healing.^[^ ^2^, ^144^, ^145^ ^]^ To address this issue, the main theory behind the self‐generation ability of EDG was the disparity in electron affinity between PTFE and Al in TENG. The most effective NPWT mode (3 s of suction followed by 1 s of inflation) was selected as the operating condition. Under this mode, the peak open‐circuit voltage reached 4.7 V, and the short‐circuit current was 100 nA. After full‐wave rectification, the maximum voltage was ≈4 V, with a current of ≈28 nA. The TENG was able to provide a robust and continuous electrical output, maintaining 92–93% of the initial open‐circuit voltage even after a 6‐day treatment cycle. During the inflammatory phase, the electric field significantly enhanced the wound's inflammatory microenvironment and modulated macrophage polarization toward the M2 phenotype. In the proliferative phase, the TENG‐generated electric field facilitated the directional migration of epidermal cells, accelerating re‐epithelialization through electrically related signaling pathways such as PI3K/Akt and MAPK/ERK. This regulation of cellular behavior is a critical rate‐limiting step in wound healing.

**Figure 5:**
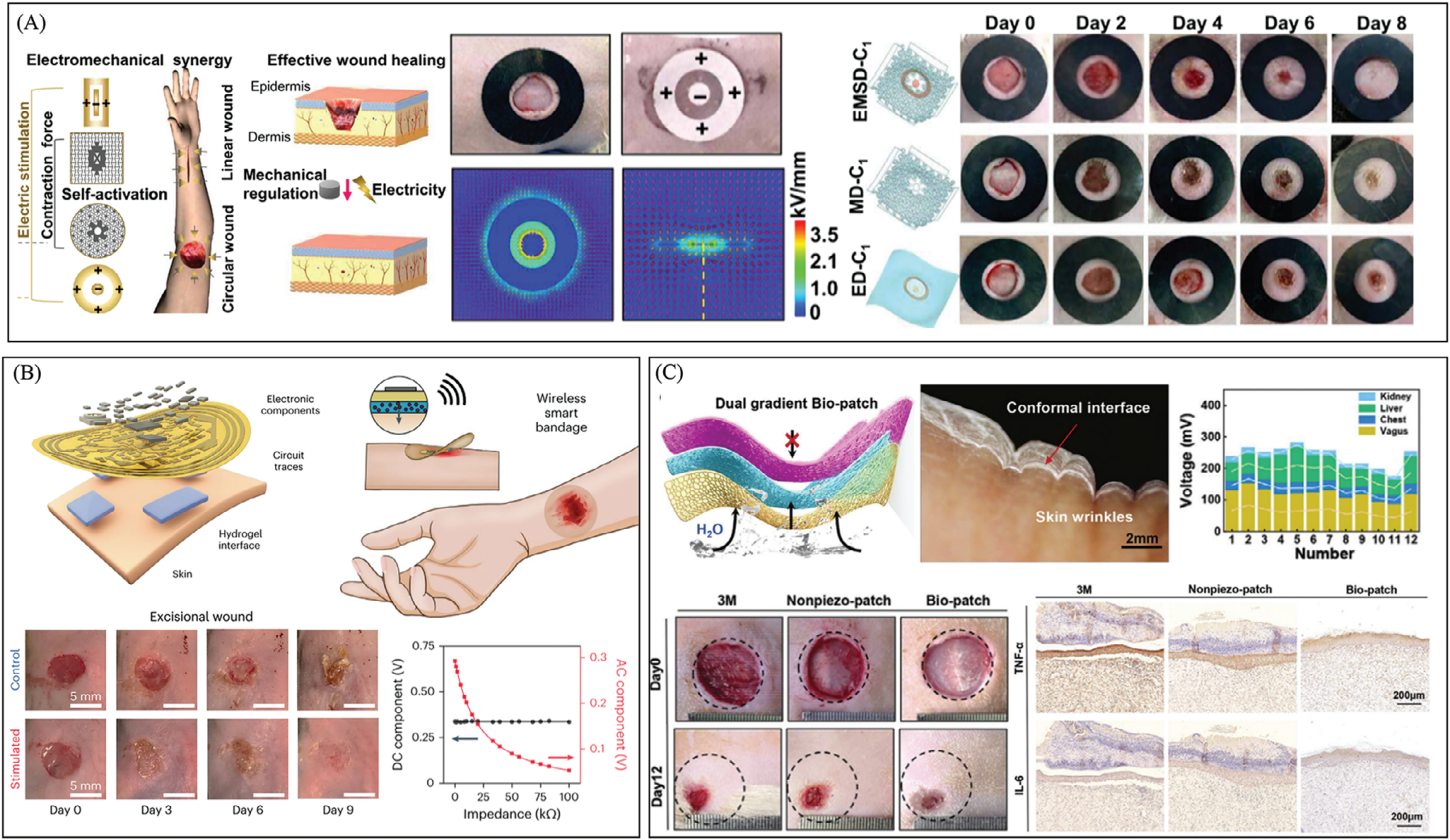
There is a growing trend in wound repair toward intelligent electrical stimulation therapy. A) A programmable and skin temperature–activated electromechanical synergistic wound dressing promotes wound healing by promoting epidermal migration and inhibiting bacterial growth through electromechanical synergistic effects. Reproduced with permission. Copyright 2022, Publisher, AAAS. B) An adjustable bioelectronic system, comprising of wirelessly driven, closed‐loop sensor and stimulation circuits, enables the early detection of infection and promotes wound healing. Reproduced with permission.^[^ ^14^ ^]^ Copyright 2022, Publisher, Springer Nature. C) A Bio‐patch that generates synchronized bionic personalized electrical signals of the vagus nerve, while promoting tissue repair by regulating macrophage polarization timing. Reproduced with permission.^[^ ^119^ ^]^ Copyright 2024, Publisher, WILEY‐VCH.

With the continuous advancement of artificial intelligence technology, electrical stimulation therapy is poised to become increasingly precise and effective for disease diagnosis and treatment. An intelligent electrical stimulation system enables personalized treatment plans by real‐time monitoring of biological signals, thereby enhancing therapeutic efficacy and improving patients’ quality of life. Yao et al. introduced a flexible and seamless electromechanical synergistic device (EMSD), composed of a shape memory alloy (SMA)‐based mechanical metamaterial grid and an antibacterial electret electrostatic film (EEF). (**Figure** **5**A) This device was designed to deliver electromechanical synergistic stimulation, effectively enhancing wound healing. Skin temperature can trigger a programmable SMA metamaterial to appropriately contract the flexible, non‐invasive EMSD, while a patterned antibacterial EEF can enhance the endogenous electric field. The Ansys Maxwell Finite Element Solver (AMFES) was utilized to estimate the electric field strength at the wound site, assessing the effectiveness of electric field penetration. The patterned electrode was found to generate a uniform electric field across its coverage area. This electric field gradually decreased to ≈0.2 V cm^−1^ at a depth of 10 mm in both the epidermal and dermal layers, which is sufficient to enhance epithelial cell migration toward the wound. When applied to the wound area, the EMSD generated a stable and effective electric field stimulation on the skin, promoting epidermal cell migration and significantly improving wound healing efficiency.

Strong electrode adhesion is essential for stable electrical signal transduction; however, maintaining high adhesion between electrodes and tissues can lead to secondary wound damage when removing adhesive dressings. So, Jiang et al. came up with a flexible bioelectronic wound care system that has a wireless power supply, closed‐loop sensing, and stimulation circuits with hydrogel electrodes that can stick to and come off of the skin as needed.^[^ ^14^ ^]^ (Figure 5B) In mice, the system demonstrated the capability to continuously monitor skin impedance and temperature, detect infections early, and adjust treatment in a closed‐loop manner to prevent further wound complications. Additionally, it provided electrical stimulation tailored to the wound environment. The treatment group showed a 25% faster healing rate and a 50% improvement in skin remodeling compared to the control group.

Recent research indicates that smart electronic patches hold significant potential for applications not only in skin tissue repair but also in the treatment of various soft tissue injuries. Additionally, TENGs have been reported for use in the treatment and prevention of atrial fibrillation.^[^ ^146^ ^]^ Qian et al. invented a personalized intelligent Bio‐patch that can interact with the vagus nerve.^[^ ^125^ ^]^ (Figure 5C) Bio‐patch achieved real‐time, non‐invasive sensing of motion through its adaptive conformability to soft tissues, seamlessly translating it into dynamic and realistic electrical signals. In a rat dorsal wound model, the Bio‐patch group demonstrated a healing rate of 95.5%, significantly outperforming the non‐piezoelectric patch group (83.5%) and exhibiting superior performance in inflammatory markers and scar thickness. Furthermore, in both rat liver injury and kidney injury models, Bio‐patch exhibited superior tissue repair and anti‐fibrosis capabilities, primarily attributed to its regulation of macrophage polarization.

#### 4.1.2 Vessel

**Figure 6:**
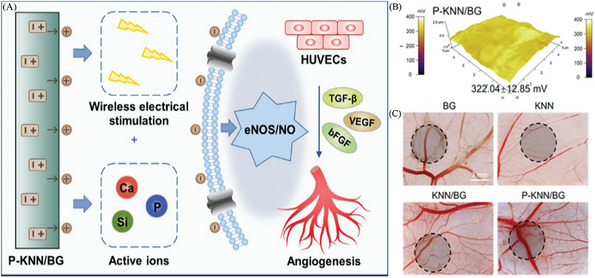
The development of electrical stimulation therapy in the field of angiogenesis. The incorporation of a composite material consisting of piezoelectric bioactive glasses (P‐KNN/BG) improves the capacity of endothelial cells to adhere and migrate, hence facilitating the process of angiogenesis. Reproduced with permission.^[^ ^120^ ^]^ Copyright 2023, Publisher, WILEY‐VCH.

Angiogenesis plays a vital role in granulation tissue formation, significantly contributing to its structure. By generating new blood vessels, angiogenesis enhances oxygenation and nutrient delivery, thereby establishing an optimal microenvironment for wound healing. It was made by Li et al. who added polarized potassium sodium niobate to a piezoelectric bioactive glass composite (P‐KNN/BG).^[^ ^120^ ^]^ (**Figure** **6**) The surface potentials of P‐KNN/BG (322.04 ± 12.85 mV) could provide excellent wireless electrical stimulation for angiogenesis. During CAM development, statistics revealed that the P‐KNN/BG group significantly increased the number, size, and length of vascular connections. The synergistic effect of radio stimulation and active ions enhanced endothelial cell adhesion and migration, demonstrating excellent angiogenic potential.

#### 4.1.3 Hair Follicle

The hair follicle is a crucial component of the skin, and its restoration is pivotal for preserving skin health and promoting recovery. Reactivating hair follicle activity significantly enhances the skin's barrier function and immune regulation. Sebum from sebaceous glands surrounding the follicle possesses antibacterial and anti‐inflammatory properties, supporting the natural balance of skin microbiota and reducing infection risk. Thus, restoring hair follicle functionality not only revitalizes the skin but also contributes to overall skin wellness.

**Figure 7:**
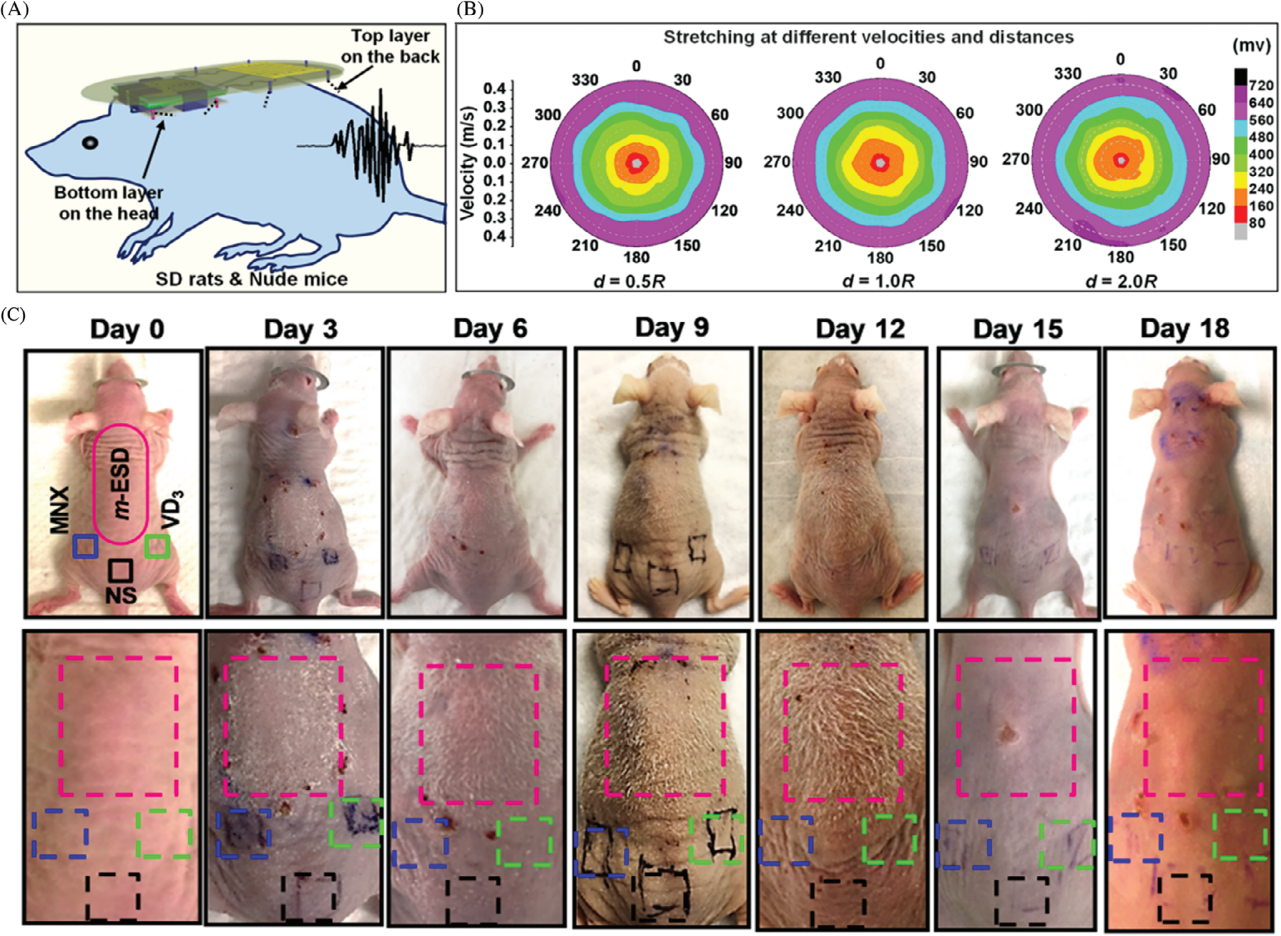
The development of electrical stimulation therapy in the field of follicle regeneration. A universal motion‐activated and wearable electric stimulation device named m‐ESD promotes hair follicle regeneration. Reproduced with permission.^[^ ^121^ ^]^ Copyright 2019, Publisher, American Chemical Society.

An alternating electric field is a non‐pharmacological physical technique that plays a crucial role in hair follicle regeneration within tissue engineering. Yao et al. developed a universal, motion‐activated, wearable electric stimulation device (m‐ESD) that effectively promotes hair regeneration through natural body movements.^[^ ^121^ ^]^ (**Figure** **7**) The peak‐to‐peak voltage (V_PP_) generated by m‐ESD was not sensitive to the distance traveled, but had a strong relationship with speed. As the speed increases from 0.05 to 0.4 m s^−1^, V_PP_ increases monotonically from 80 to 720 mV. m‐ESD could generate stable voltage pulses in response to random lateral movements, making it an ideal choice for integration with natural body motion. The results of Yang et al.’s showed that microneedle‐based self‐powered transcutaneous electrical stimulation system (mn‐STESS) also had the potential to promote hair follicle growth.^[^ ^24^ ^]^ (Figure 4A) Electricity as an electrical adjuvant to promote hair follicle regeneration might be a potential development direction for future electrical stimulation therapy.

### 4.2 Nerve Repairing

The significance of nerve repair in plastic surgery tissue transplantation is in its ability to improve the regeneration and functional restoration of transplanted muscles and tissues, while additionally enabling the provision of nutrients and control of metabolism.^[^ ^147^, ^148^ ^]^ Damaged nervous systems frequently result in functional impairments in associated muscle tissues, including muscle atrophy and weakness.^[^ ^149^, ^150^ ^]^ Nerve regeneration establish a pathway for nerve impulse transmission within damaged muscle tissues, enhancing the transmission of sensory stimuli and the control of muscle contractions by nerve endings, particularly in areas requiring precise actions, such as the hands and face. Nerve repair and tissue transplantation can restore these functions, enabling patients to regain precise movements and sensory awareness.^[^ ^151^ ^]^ In the adult tissue microenvironment of the central nervous system (CNS), the inherently poor regenerative capacity of neurons and the presence of inhibitory factors are major obstacles to axonal regeneration and functional reconstruction. In the peripheral nervous system (PNS), although it possesses relatively stronger self‐repair abilities, permanent neurological deficits often persist, accompanied by failed nerve regeneration and the occurrence of chronic pain.^[^ ^152^, ^153^ ^]^ Currently, stem cell differentiation, myelination, and axonal extension primarily induce neural repair. Stem cell differentiation is one of the key pathways for neural regeneration. Stem cells possess the ability for self‐renewal and multipotent differentiation, enabling them to differentiate into neurons, glial cells, and other neural support cells.^[^ ^154^, ^155^ ^]^ Myelination and axonal extension are essential for neural regeneration, with glial cells wrapping myelin sheaths around axons to enhance signal transmission—these processes collectively support neural repair. As nerves are electrogenic cells, electrical stimulation is frequently used in the treatment of nerve injury‐related diseases.^[^ ^151^ ^]^

**Figure 8:**
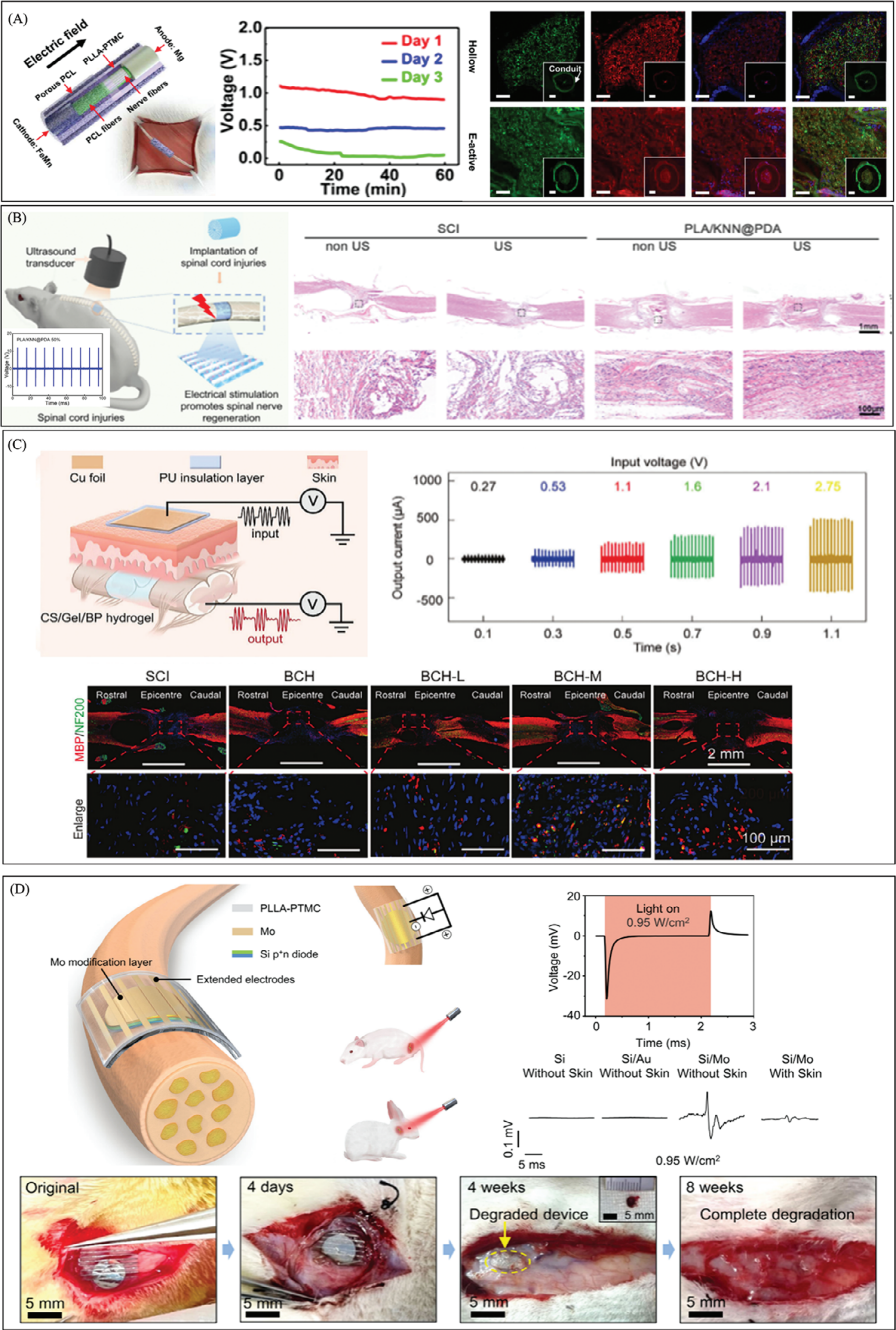
The development of electrical stimulation therapy in the field of nerve repair. A) A fully biodegradable, self‐electrified, and miniaturized device made up of dissolvable galvanic cells on a biodegradable scaffold helps nerve regeneration by increasing calcium activity and axonal growth. Reproduced with permission.^[^ ^122^ ^]^ Copyright 2020, Publisher, AAAS. B) A wireless electrical stimulation 3D piezoelectric scaffold that is biodegradable and high‐performance and is driven by ultrasound (US) helps neural stem cells differentiate and helps the spinal cord heal. Reproduced with permission.^[^ ^123^ ^]^ Copyright 2022, Publisher, American Chemical Society. C) A conductive hydrogel that can generate power on‐site can be used as a biodegradable regenerative scaffold and a wireless electrical stimulation platform to help the myelin sheath grow back and nerves heal. Reproduced with permission.^[^ ^124^ ^]^ Copyright 2024, Publisher, WILEY‐VCH. D) A biodegradable and flexible neural interface for transdermal optoelectronic modulation and regeneration of peripheral nerves. Reproduced with permission.^[^ ^125^ ^]^ Copyright 2024, Publisher, Springer Nature.

Electrical stimulation is an effective strategy for treating neural disorders, leveraging the intrinsic electrical responsiveness of nerve cells. This responsiveness encompasses ion channel activity on the cell membrane, membrane potential changes, action potential propagation, and synaptic transmission. The ability of nerve cells to respond to electrical stimuli plays a critical role in neural signaling, functional regulation, and pathophysiological processes, making electrical stimulation a promising approach for promoting neuronal regeneration. Wang et al. created a small, biodegradable primary battery that connects the original cell to the nerve guide catheter. This gave structural support and self‐sustaining electrical stimulation outside of the surgery window to help peripheral nerve regeneration.^[^ ^122^ ^]^ (**Figure** **8**A) The electroactive device delivered continuous electrical stimulation beyond the intraoperative period. Its key electroactive component, the Mg‐FeMn primary battery, significantly enhanced axonal growth and calcium activity in DRG neurons, subsequently promoting Schwann cell proliferation and neurotrophic factor production. The device's self‐powered nature eliminates the need for external power sources, ensuring ease of use.

In order to get rid of the limitation of the battery capacity of the device volume, Chen et al. designed a biodegradable high‐performance 3D piezoelectric scaffold with wireless electrical stimulation function driven by ultrasound (US).^[^ ^123^ ^]^ (Figure 8B) Once implanted in the body, the US‐driven nanogenerator achieves a V_oc_ of ≈11.56 V at an acoustic pressure of 150 kPa. There are several slight attenuations compared to the 12.09 V observed in vitro. Under appropriate US stimulation, it was verified in a laboratory setting that the neural differentiation of neural stem cells (NSCs) was enhanced. Observations in a rat model of complete spinal cord injury demonstrated significant improvement in motor function recovery and spinal cord repair. This provided evidence that the wireless device may effectively contribute to in vivo energy supply.

Wu et al. demonstrated a conductive hydrogel that can generate electricity in‐situ, serving as a biodegradable scaffold for tissue regeneration and a wireless electrical stimulation platform.^[^ ^124^ ^]^ (Figure 8C) When an insulating metal plate is placed on top of the damaged area as a wireless power transmitter, due to electrostatic induction effects, capacitive coupling between the receiver and transmitter at the damaged area can generate AC power in the hydrogel scaffold. The desired electrical stimulation output can be obtained by adjusting the input voltage applied to the insulated copper foil, and the output current and output voltage are proportional to it. In the rat model of total spinal cord injury, this self‐generating scaffold could promote neural recovery by increasing myelin regeneration, speeding axonal regeneration, and stimulating the differentiation of endogenous neural stem cells.

Sun et al. introduced a compact and flexible optoelectronic device made of thin‐film silicon diodes that is biodegradable. This device allows wireless transdermal stimulation and promotes the restoration of peripheral nerves. It has been successfully tested on rabbits with facial nerve injuries.^[^ ^125^ ^]^ (Figure 8D) As shown in the figure, in the rabbit model of facial nerve injury, the flexible optoelectronic device maintained good adhesion and degradability, reducing the harm of secondary surgery and related infection risks. By further optimizing the equipment, the flexible optoelectronic device can also be used to treat other nerve ailments, such as those affecting the vagus nerve. This information is highly informative for the clinical management of plastic surgery.

### 4.3 Locomotor System Recovery

The regeneration of bone, cartilage, and tendon is essential in plastic surgery repair.^[^ ^156^, ^157^ ^]^ These tissues are fundamental to the musculoskeletal system, providing support, force transmission, and joint stability.^[^ ^158^ ^]^ Bone regeneration aids in fracture healing, defect repair, and disease treatment, restoring skeletal integrity and joint mobility.^[^ ^159^ ^]^ Cartilage, as the protective tissue covering joint surfaces, reduces friction and absorbs impact,^[^ ^160^, ^161^ ^]^ its regeneration alleviates joint discomfort, enhances stability, and increases mobility.^[^ ^162^ ^]^ Tendons, which connect muscles to bones and transmit contraction forces, are crucial for movement; tendon regeneration strengthens load‐bearing capacity and flexibility, thereby improving motor coordination.^[^ ^163^ ^]^

#### 4.3.1 Bone

The bioelectromotive force (BMF) of natural bone is essential for regulating cellular metabolism, including growth, proliferation, differentiation, and migration, all of which are fundamental to processes like fracture healing.^[^ ^164^ ^]^ For example, the BMF generated during human tibial walking is ≈300 µV.^[^ ^165^, ^166^ ^]^ The intraosseous BMF enhances the generation of negative charges under physiological compressive loads, stimulating osteoblast activity and accelerating matrix mineralization at fracture sites.^[^ ^112^ ^]^ Consequently, piezoelectric stimulation is crucial in regulating bone regeneration. Beyond the piezoelectric effect, bone regeneration can be further enhanced through direct electrical stimulation.^[^ ^167^, ^168^ ^]^

**Figure 9:**
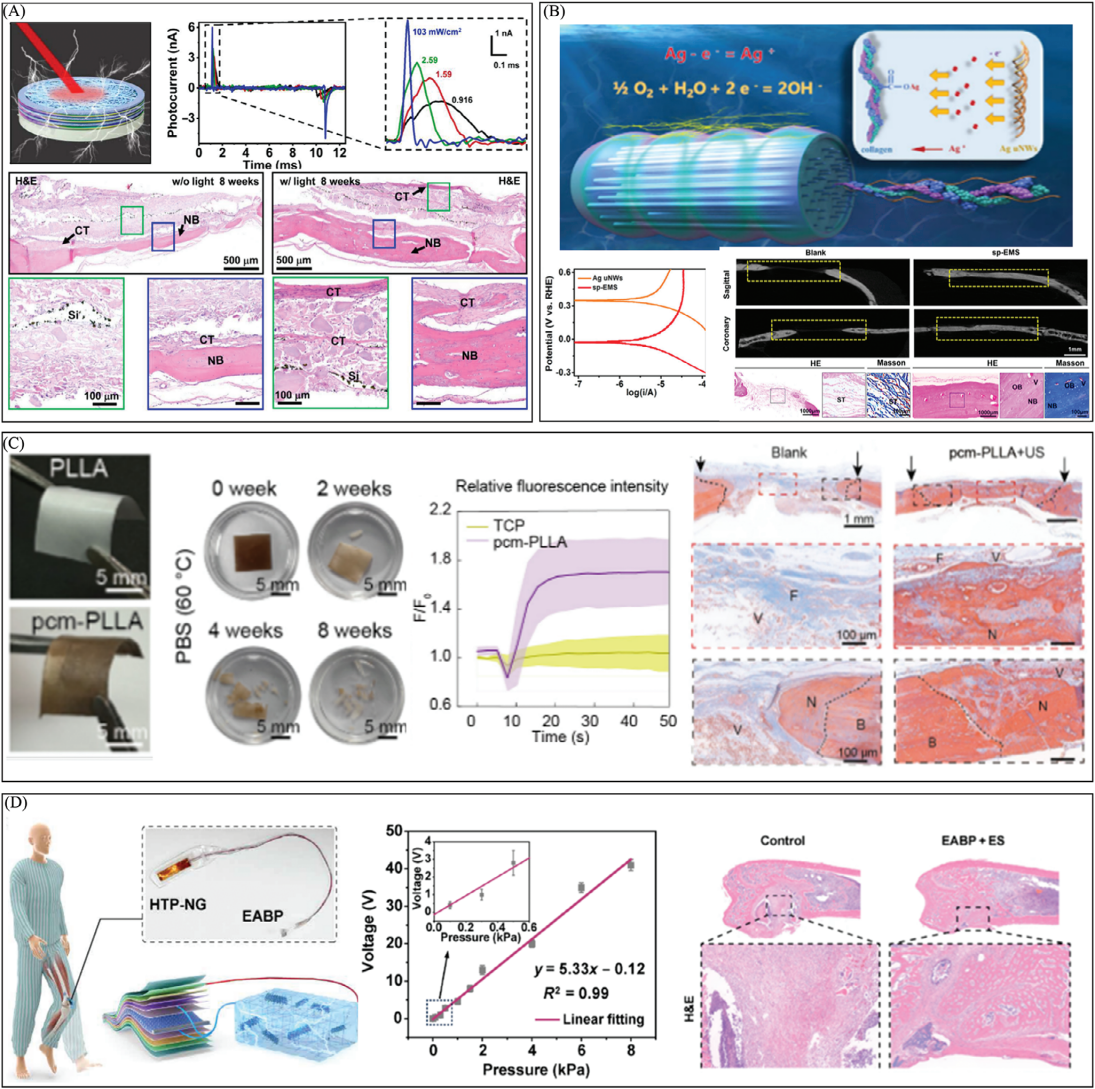
The development of electrical stimulation therapy in the field of bone regeneration. A) A 3D biomimetic scaffold combined with thin‐film silicon (Si)‐based microstructures helps cells grow and differentiate so that bone can be repaired. Reproduced with permission.^[^ ^23^ ^]^ Copyright 2023, Publisher, AAAS. B) A self‐promoted electroactive mineralized scaffold (sp‐EMS) that creates weak electrical signals through electrochemical reactions on its own helps fight bacteria and bone regeneration. Reproduced with permission.^[^ ^126^ ^]^ Copyright 2023, Publisher, Springer Nature. C) A tissue engineering scaffold (pcm‐PLLA) creates an environment that is like bone through a piezocatalytically‐induced controllable mineralization strategy. This creates a better immune microenvironment and increases stem cells to help bone formation. Reproduced with permission.^[^ ^18^ ^]^ Copyright 2024, Publisher, Elsevier. D) A hybrid friction/PENG that sends out biphasic electrical pulses during rehabilitation training. This creates synergistic effects of electrical and mechanical stimulation, which raises the expression of different proteins that help bones grow back. Reproduced with permission.^[^ ^127^ ^]^ Copyright 2024, Publisher, Springer Nature.

The clinical treatment of refractory, delayed, and non‐healing fractures has widely adopted the use of external electrical stimulation to stimulate the endogenous electric field of bone tissue, thereby accelerating bone regeneration. Wang et al. showed a 3D biomimetic scaffold made of thin‐film silicon (Si) microstructures that can control the membrane potential and intracellular calcium dynamics of stem cells using an electrical signal. This helps the cells divide and grow.^[^ ^23^ ^]^ (**Figure** **9**A) Animal experiment results showed that the bone density of the experimental group increased, and the regeneration of the skull was more complete.

Orthopedic treatment urgently needs to address infectious bone defects in addition to refractory fractures. However, antibiotics not only struggle to address complex infections but also negatively impact the regeneration of bone grafts. To solve this problem, Li et al. created a self‐promoted electroactive mineralized scaffold (sp‐EMS) that could make weak electrical signals by using electrochemical reactions that happened on their own.^[^ ^126^ ^]^ (Figure 9B) Three animal models were employed in this study to evaluate the material's efficacy in treating bone infections: a rat skull defect with a single bacterial infection, a rabbit open alveolar defect within a complex oral bacterial microenvironment, and a beagle dog vertical bone defect. Across all models, the scaffolds demonstrated remarkable infection resistance and promoted bone regeneration.

The natural bone microenvironment maintains a balance of mechanical, chemical, and electrical properties. The self‐assembly of the biopolymer collagen and inorganic hydroxyapatite (HA) forms a hierarchical structure, imparting significant mechanical strength to bones. The asymmetric molecular structure of collagen fibers grants bones their unique piezoelectric properties. This balanced microenvironment is essential for bone growth and development. Cui et al. developed a tissue engineering scaffold named pcm‐PLLA, designed to mimic the bone‐like microenvironment. This was achieved by a regulated mineralization process caused by piezocatalysis, using the degradable piezoelectric polymer poly‐L‐lactic acid (PLLA).^[^ ^18^ ^]^ (Figure 9C) In vivo experiments demonstrated significant stem cell accumulation within the pc‐PLLA scaffold, along with a reduced M1/M2 macrophage ratio and enhanced angiogenesis. This scaffold rapidly attracts stem cells during the initial stages of bone repair, enhances the immune microenvironment at the bone defect site, lays the groundwork for subsequent bone repairs, and significantly reduces the time required for bone defect treatment. Enabling the opening of voltage‐gated Ca^2+^ channels in the cell membrane results in a substantial increase in intracellular Ca^2+^ influx, which subsequently accelerates osteogenic differentiation.

Beyond bone defect repair, the patient's overall experience is profoundly shaped by functional recovery achieved through rehabilitation training. Recently, researchers in the field of skeletal muscle injury have discovered biofeedback ES therapy, which associates stimulation parameters with dynamic movement, resulting in more significant therapeutic effects than traditional electrical stimulation. It is not difficult to imagine that it may also have the same effect in bone regeneration. Wang et al. described a bone defect electrical stimulation (BD‐ES) system that could be fully implanted. It had a hybrid friction/PENG (HTP‐NG) that could provide biphasic electrical pulses during rehabilitation training using bioactive conductive gels.^[^ ^127^ ^]^ (Figure 9D) In the rat femoral defect model, the BD‐ES system showed good bone regeneration performance. The BD‐ES system demonstrated the ability to enhance protein expression in response to mechanical stimulation via the PI3K/AKT, WNT, and MAPK signaling pathways. Specifically, GSEA analysis revealed an upregulation of mechanosensitive proteins PIEZO1 and PIEZO2. These findings suggest that electrical stimulation may synergize with mechanical stimulation to modulate protein expression, thereby supporting bone regeneration.

#### 4.3.2 Cartilage

Given the ubiquity of bioelectric signals within the body, electrical stimulation emerges as a promising approach for tissue regeneration, particularly in cartilage, which is highly responsive to electrical stimulation. Researchers have utilized battery and bimetallic electrodes to generate nanoampere currents, effectively stimulating hyaline cartilage growth in rabbit knees. Direct currents have been applied to support cartilage repair, while biphasic currents have demonstrated efficacy in promoting hyaline cartilage regeneration in male rats.

**Figure 10:**
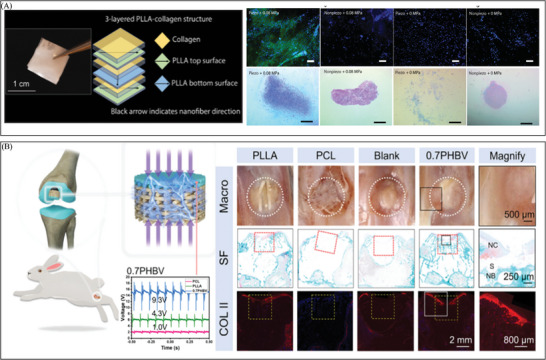
The development of electrical stimulation therapy in the field of cartilage regeneration. A) To promote cartilage formation and regeneration under external forces, a biodegradable piezoelectric polylactic acid (PLLA) nanofiber scaffold serves as a battery‐free electrical stimulator. Reproduced with permission.^[^ ^128^ ^]^ Copyright 2022, Publisher, AAAS. B) A new biodegradable composite (70% PHBV and 30% PLLA, wt.%) called 0.7PHBV has a lower modulus and a higher piezoelectricity. It turns on calcium and TGF‐b signaling pathways to help cartilage grow back. Reproduced with permission.^[^ ^129^ ^]^ Copyright 2024, Publisher, Elsevier.

In order to stimulate cartilage formation and regeneration under external forces, Liu et al. designed a biodegradable piezoelectric polylactic acid (PLLA) nanofiber scaffold.^[^ ^128^ ^]^ (**Figure** **10**A) The researchers discovered that the non‐degradable piezoelectric polyvinylidene fluoride‐trifluoroethylene (PVDF‐TrFE) with a low output voltage (≈20 mV mm^−1^) helped cartilage differentiate. The voltage that this scaffold produced was also in the right range for cartilage growth. It can successfully improve the chondrogenic differentiation of stem cells in the lab and help cartilage grow back in a rabbit knee model of osteoarthritis (OA) with critical‐size osteochondral (OC) defects.

Poly(l‐lactide) (PLLA) is a promising piezoelectric biomaterial with potential applications in tissue repair. However, its low piezoelectric coefficient significantly limits its effectiveness for in situ piezoelectric stimulation therapy. Herein, Li et al. developed a new kind of biodegradable composite (70% PHBV and 30% PLLA, wt.%), termed 0.7 PHBV, which had reduced modulus and increased piezoelectricity, with a piezoelectric coefficient 2.0‐2.5 times that of PLLA.^[^ ^129^ ^]^ (Figure 10B) In the rabbit cartilage defect model, the material showed beneficial effects in promoting cartilage regeneration. Bioinformatics analysis revealed that the piezoelectric scaffold may help cartilage grow back by turning on the calcium and TGF‐β signaling pathways, which creates cartilage.

#### 4.3.3 Tendon

**Figure 11:**
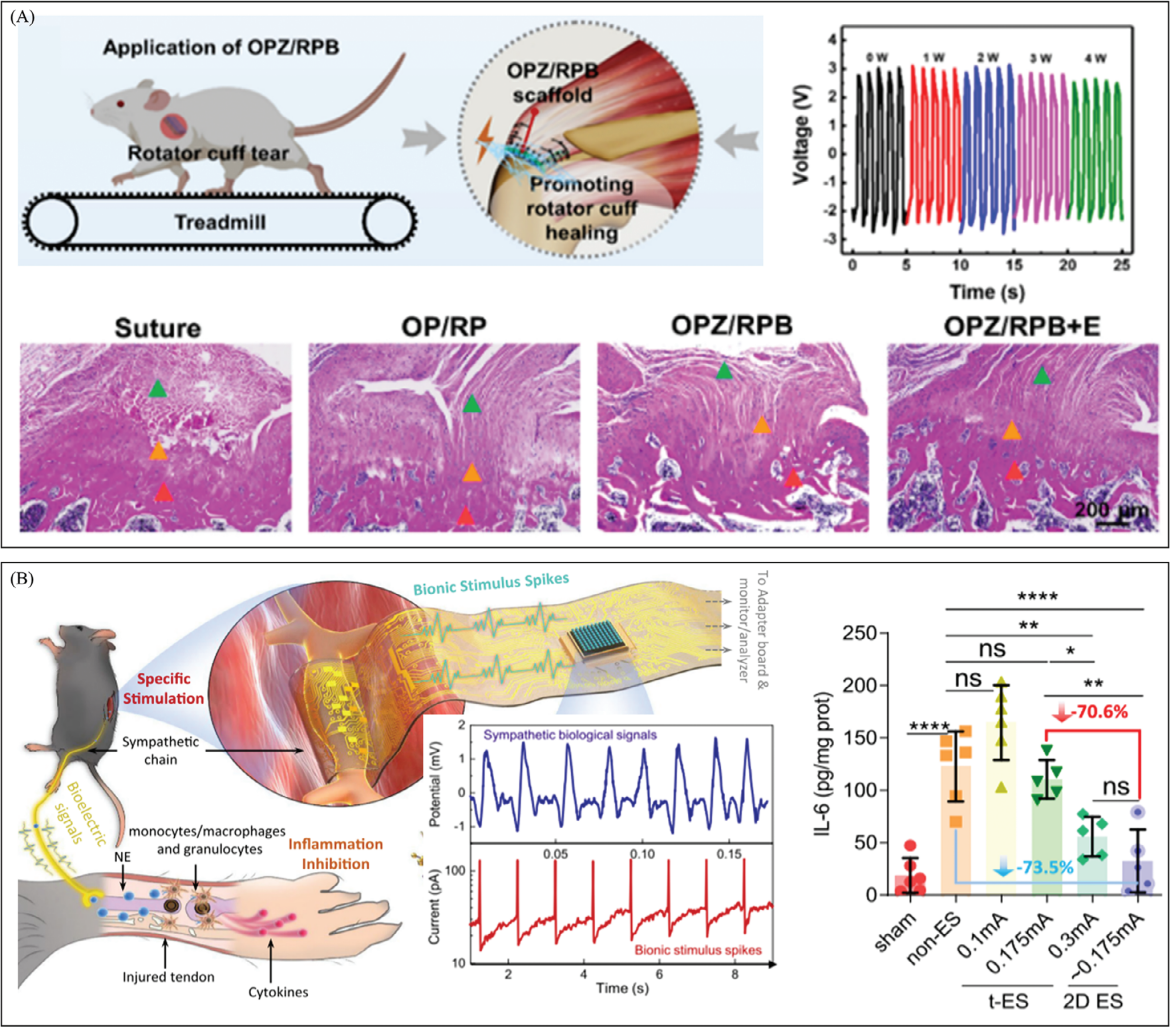
The development of electrical stimulation therapy in the field of tendon regeneration. A) To promote tendon‐bone healing, a Janus nanofiber scaffold with piezoelectric properties and a biomimetic collagen fiber arrangement produces electrical stimulation in conjunction with rehabilitation training. Reproduced with permission.^[^ ^130^ ^]^ Copyright 2024, Publisher, Elsevier. B) The neuromorphic electrical stimulation is based on an atomically thin MoS2 floating gate memory (FGM) digital intercircuit (IDC), and it uses flexible electrodes wrapped around the sympathetic chain for direct stimulation to inhibit acute inflammation that leads to tendon adhesion. Reproduced with permission.^[^ ^131^ ^]^ Copyright 2024, Publisher, Elsevier.

Besides bone and cartilage, there is less research on tendon repair, mainly focusing on tendon‐bone healing.^[^ ^169^ ^]^ Zhang et al. evaluated the effect of electrical stimulation on tendon rupture repair, they showed a Janus nanofiber scaffolds with piezoelectric properties and biomimetic collagen fiber arrangement prepared by electrospinning back‐to‐back layers of poly (L‐lactic acid)/zinc oxide (PLLA/ZnO) and PLLA/barium titanate (PLLA/BTO).^[^ ^130^ ^]^ (**Figure** **11**A) The treatment model for tendon sheath tears in rats demonstrated that the Janus nanofibrous piezoelectric scaffold (OPZ/RPB), when combined with exercise training, provide mechanical and electrical stimulation, which in turn promotes the concurrent healing of the tendon‐bone interface, leading to improved histological and biomechanical outcomes.

Peripheral nerve electrical stimulation offers an alternative approach to treating inflammation, distinct from direct material implantation into the tendon. This method modulates bioelectric signals and neurotransmitter activity, thereby influencing inflammatory cell behavior and effectively reducing inflammation. In this study, Bao et al. showed a neuromorphic electrical stimulation that is based on an atomically thin MoS_2_ floating gate memory (FGM) digital intercircuit (IDC). They used flexible electrodes that were wrapped around the sympathetic chain to directly stimulate it and stop the acute inflammation that causes tendon adhesion.^[^ ^131^ ^]^ (Figure 11B) MoS_2_ FGM can be programmed in a way that is similar to biological synapses. This means that it can act like biological synapses that change over time and create neuromorphic bionic spikes. Direct emission of electrical stimulation to the nerve segments innervating the tendon results in a 73.5% decrease in cytokine IL‐6 in 2D electrical stimulation mice compared to non‐ electrical stimulation mice, indicating the considerable promise of this material in treating tendon injuries.

**Figure 12:**
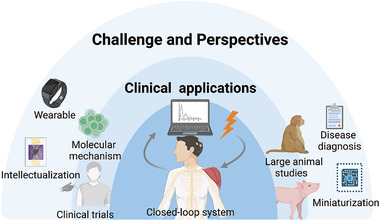
Schematic illustration of conclusions and perspectives about electrical stimulation therapy for plastic repair.

## 5 Discussion and Outlook

The development of electrical stimulation therapy in plastic surgery has transitioned from bulky, externally powered, wired, and non‐degradable devices to compact, self‐generating, wireless, and biodegradable systems. This evolution has refined and personalized treatment options, enhancing patient outcomes and improving comfort, while pushing the boundaries of plastic surgery innovation (Figure 12).

Initially, early electrical stimulation devices were large and dependent on external power sources, which limited patient mobility an convenience.^[^ ^170^, ^171^ ^]^ Advances in technology have introduced smaller, portable, and battery‐operated devices, greatly improving both safety and therapeutic efficacy.^[^ ^172^, ^173^ ^]^

A significant advancement in recent years has been the transition from externally powered devices to self‐generating technologies, such as nanogenerators and electromagnetic induction coils, which harness the patient's physiological activity or external energy sources, offering flexibility and convenience in treatment delivery.^[^ ^174^, ^175^ ^]^

Additionally, the shift to biodegradable materials in electrical stimulation devices has reduced the need for device removal surgeries, minimizing patient discomfort. These modern devices naturally degrade and absorb within the body, decreasing the risks associated with long‐term implant retention and enhancing patient quality of life.^[^ ^176^ ^]^

In plastic surgery, electrical stimulation therapy has also evolved from a single therapeutic approach to a more integrated treatment modality. Traditionally, single‐mode electrical stimulation was applied for conditions such as dysfunction, muscle atrophy, and chronic wounds.^[^ ^48^, ^177^ ^]^ While effective, the demand for better outcomes has led to the development of combined electrical stimulation therapy, which integrates electrical stimulation with other treatments such as chemotherapy, physical therapy, and rehabilitation exercises.^[^ ^26^, ^150^, ^151^ ^]^

In skin regeneration, electrical stimulation can enhance drug release stability and be combined with negative pressure therapy to promote tissue regeneration and reduce inflammation.^[^ ^24^, ^178^ ^]^ For motor system rehabilitation, combining electrical stimulation with rehabilitation exercises in biofeedback ES therapy links stimulation parameters with dynamic movements, resulting in better therapeutic effects and improved bone regeneration and functional recovery.

This multidisciplinary approach leverages various treatments to achieve synergistic effects that enhance patient outcomes and quality of life, opening new possibilities for clinical treatment and research in plastic surgery. The development of combined treatment strategies presents both challenges and opportunities. Determining the optimal combination of therapies, understanding their synergistic effects and mutual influences, and ensuring safety and effectiveness require further in‐depth research.

Furthermore, this approach provides new opportunities for developing innovative treatment devices and technologies. For example, advancements in wearable and intelligent systems can enhance therapeutic outcomes.^[^ ^179^ ^]^ In multi‐tissue regeneration, electrical stimulation is crucial for promoting the repair and regeneration of skin, blood vessels, nerves, and bone. By integrating essential biological processes—including keratinocyte migration, angiogenesis, neural repair, and osteogenesis—combined treatments bring significant progress to plastic surgery. These integrated approaches not only elevate patient outcomes but also drive deeper investigation into the underlying mechanisms of tissue regeneration, paving the way for more effective and holistic clinical applications in plastic surgery and regenerative medicine.^[^ ^180^ ^]^

### 5.1 Optimizing the Performance

#### 5.1.1 Closed‐Loop System

In precision medicine, an interactive, closed‐loop diagnostic and therapeutic model is essential for disease identification and patient‐specific management. This system enables real‐time monitoring of the tissue microenvironment, offering immediate feedback on changes in tissue status and autonomously adjusting electrical stimulation parameters to align with the individualized needs of each patient. Integrating embedded sensors, intelligent feedback systems, and electrical stimulation therapy into closed‐loop models is essential for advancing innovative, stimulation‐based repair techniques in fields like orthopedic and reconstructive surgery.

#### 5.1.2 Long‐Term Biocompatibility

Achieving safe and effective clinical outcomes requires electrical stimulation devices to possess high levels of biocompatibility, particularly for long‐term implantation. Long‐term biocompatibility is essential to the sustained efficacy of implantable electrodes and electrical stimulation devices. Selecting materials with excellent biocompatibility, mechanical compatibility, and optimized encapsulation ensures that implanted devices can remain stable and function effectively over extended periods in vivo, minimizing risks of adverse reactions.

#### 5.1.3 Equipment Miniaturization

For wearable electrical stimulation devices, portability and ease of use are essential, while implantable devices benefit from miniaturization, which reduces the risk of tissue rejection and enhances comfort. Utilizing micro‐electrodes enables precise electrical stimulation therapy. Therefore, medical electrical stimulation devices must be lightweight and miniaturized, capable of delivering accurate and reliable stimulation across diverse clinical environments.

### 5.2 Advancing Clinical Trials

#### 5.2.1 Establishing Guidelines for Electrical Stimulation Therapy

In plastic surgery, electrical stimulation therapy is a widely used technique to promote tissue repair. However, the standardization of electrical stimulation therapy remains a significant challenge. Specific guidelines are lacking in several key areas: 1) Defining consistent evaluation criteria for the effectiveness of electrical stimulation therapy for the same condition; 2) Developing treatment protocols tailored to different types of diseases; 3) Standardizing therapy parameters across diverse populations, including variations in age, gender, and body weight; and 4) Establishing practical application standards for wearable and implantable electrical stimulation devices.^[^ ^181^, ^182^ ^]^

To address these challenges, future research should consider the following strategies: 1) Clearly identify conditions appropriate for electrical stimulation therapy, further classifying them into subtypes and progression stages; this involves defining disease stages using animal models and conducting rigorous validation studies; 2) Collaborate with clinical experts in plastic surgery to develop standardized protocols, including specific electrical stimulation parameters and evaluation criteria for therapeutic outcomes across various conditions.

#### 5.2.2 Initiating Large Animal Studies

Current research in electrical stimulation therapy primarily relies on small animal models, such as rats and rabbits, with limited studies on larger animals, like pigs and dogs. Given the physiological similarities of pigs, dogs, and primates to humans, expanding research to larger animal models could enhance our understanding of the therapy's efficacy and biosafety. This approach will provide valuable insights for future human clinical trials, bridging preclinical findings to practical human applications.

#### 5.2.3 Launching Human Clinical Trials

Launching human clinical trials represents a pivotal step in the field of electrical stimulation therapy. Most current devices for this therapy are non‐invasive, making them suitable for a broad patient population, including individuals with chronic wounds and motor disorders. Conducting human trials will allow for incremental improvements in therapy attributes and facilitate the integration of electrical stimulation as a mainstream treatment option in plastic surgery.

## Conflict of Interest

The authors declare no conflict of interest.
